# Formulation and Evaluation of Propranolol Hydrochloride-Loaded Carbopol-934P/Ethyl Cellulose Mucoadhesive Microspheres

**Published:** 2010

**Authors:** Jayvadan Patel, Darshna Patel, Jignyasha Raval

**Affiliations:** a *Nootan Pharmacy College, Visnagar-384315, Gujarat, India*.; b *S. K. Patel College of Pharmaceutical Education and Research, Ganpat University, Kherva-382711, Gujarat, India.*

**Keywords:** Mucoadhesive, Propranolol hydrochloride, Microspheres, Factorial design, Anti-hypertensive

## Abstract

The purpose of this research was to formulate and systemically evaluate *in-vitro *and *in-vivo *performances of mucoadhesive propranolol hydrochloride microspheres for its potential use in the treatment of hypertension, myocardial infraction and cardiac arrhythmias. Propranolol hydrochloride mucoadhesive microspheres, containing carbopol-934P as mucoadhesive polymer and ethyl cellulose as carrier polymer, were prepared by an emulsion-solvent evaporation technique. Results of preliminary trials indicated that the quantity of emulsifying agent, time for stirring, drug-to-polymers ratio, and speed of rotation affected various characteristics of microspheres. Microspheres were discrete, spherical, free-flowing and showed a good percentage of drug entrapment efficiency. An *in-vitro *mucoadhesive test showed that propranolol hydrochloride mucoadhesive microspheres adhered more strongly to the gastric mucous layer and could be retained in the gastrointestinal tract for an extended period of time. A 3^2^ full factorial design was employed to study the effect of independent variables, drug-to-polymer-to-polymer ratio (propranolol hydrochloride-ethyl cellulose-carbopol-934P) (*X*_1_), and stirring speed (*X*_2_) on dependent variables, i.e. percentage of mucoadhesion, drug entrapment efficiency, particle size and t_80_. The best batch exhibited a high drug entrapment efficiency of 54 %; 82% mucoadhesion after 1 h and particle size of 110 μm. A sustained pattern of drug release was obtained for more than 12 h. The drug-to-polymer-to-polymer ratio had a more significant effect on the dependent variables. The morphological characteristics of the mucoadhesive microspheres were studied under a scanning electron microscope. *In-vivo *evaluation studies on propranolol hydrochloride mucoadhesive microspheres and propranolol hydrochloride powder were performed on normal healthy rabbits. The results showed a sustained anti-hypertensive effect over a longer period of time in case of mucoadhesive microspheres, compared to the powder. In conclusion, the prolonged gastrointestinal residence time and slow release of propranolol hydrochloride resulting from the mucoadhesive microspheres, could contribute to the provision of a sustained anti-hypertensive effect.

## Introduction

Microsphere carrier systems made from the naturally occurring biodegradable polymers have attracted considerable attention for several years in sustained release drug delivery. Recently, dosage forms that can precisely control the release rates and target drugs to a specific body site have made an enormous impact in the formulation and development of novel drug delivery systems. Microspheres form an important part of such novel drug delivery systems (1-3). They have varied applications and are prepared using assorted polymers (4). However, the success of these microspheres is limited owing to their short residence time at the site of absorption. It would, therefore, be advantageous to have means for providing an intimate contact of the drug delivery system with the absorbing membranes (5-8). This can be achieved by coupling mucoadhesion characteristics to microspheres and developing mucoadhesive microspheres. Mucoadhesive microspheres have advantages such as efficient absorption and enhanced bioavailability of drugs owing to a high surface-to-volume ratio, a much more intimate contact with the mucus layer, and specific targeting of drugs to the absorption site (9-12). Carbopol-934P (acrylic acid homopolymer) is an anionic polymer that has been used in mucoadhesive systems by several researchers (13-17). Carbopol-934P has been selected as a polymer in the preparation of mucoadhesive microspheres because of its good mucoadhesive properties and is not absorbed by body tissues and being totally safe for human oral consumption.

Propranolol hydrochloride, a beta-adrenorecptor antagonist that can acutely lower the blood pressure in human by blocking receptors non-selectively, is typically prescribed to treat hypertension, myocardial infraction and cardiac arrhythmias. Its short biological half-life (3.9 ± 0.4 h) necessitates the need for its administration in two or three dosage forms of 40 to 80 mg per day (18). Thus, the development of controlled-release dosage forms would clearly be advantageous. Researchers have formulated oral controlled-release products of propranolol hydrochloride by various techniques (19-21). Moreover, the site of absorption of propranolol hydrochloride is in the stomach. Dosage forms that are retained in the stomach would increase the absorption, improve drug efficiency, and decrease dose requirements. 

In context of the above principles, a strong need was felt to develop a dosage form that delivered propranolol hydrochloride into the stomach and would increase the efficiency of the drug, providing a sustained action. Thus, an attempt was made in the present investigation to use Carbopol-934P as a mucoadhesive polymer and ethyl cellulose as carrier polymer, in order to prepare mucoadhesive propranolol hydrochloride microspheres. The microspheres were characterized by in-vitro and iv-vivo tests and factorial design was used to optimize the variables. 

## Experimental

*Materials*

Propranolol hydrochloride (powder) was obtained as a gift sample from Zydus Cadila (Ahmedabad, India). Carbopol-934P (CP, molecular weight of 3 × 10^6 ^Da,) was obtained as a gift sample from Noveon^® ^(Mumbai, India). Ethyl cellulose (50 cps viscosity standard grade) and petroleum ether 80:20 were procured from Willson Lab (Mumbai, India) and S. D. Fine Chemicals Ltd. (Mumbai, India), respectively. Liquid paraffin and span 80 were purchased from Loba Chemie Pvt Ltd. (Mumbai, India). All other ingredients were of analytical grade.

*Animals*

Six-months-old mixed sex, specific pathogen-free healthy Indian white rabbits (lupas) (Body weight 2.3 to 2.65 kg), were gifted from Zydus Cadila (Ahmedabad, India) and maintained under standard laboratory conditions (room temperature, 23 ± 2°C; relative humidity, 55 ± 5%; 12/12 h light/dark cycle) with free access to a commercial rodent diet and tap water.

*Preparation of mucoadhesive propranolol hydrochloride microspheres*

Mucoadhesive propranolol hydrochloride microspheres were prepared containing Carbopol-934P as the mucoadhesive polymer and ethyl cellulose as the carrier polymer, by emulsion-solvent evaporation technique. Briefly, ethyl cellulose (1500 mg) was dissolved in 200 mL of ethanol. Each 500 mg of propranolol hydrochloride and Carbopol-934P were dispersed in the ethyl cellulose solution, of under stirring. In preliminary trail batches, the drug-to-polymer-to-polymer (propranolol hydrochloride-ethyl cellulose-Carbopol-934P) ratio was kept constant at 1:3:1. The resultant mixture was extruded through a syringe (No. 20) in 500 mL of liquid paraffin (heavy and light, 1:1 ratio) containing 2.0 % v/v Span 80 and stirring was carried out using a propeller stirrer (Remi, Mumbai, India) at 1000 rpm. Stirring was continued for 3 h. The amount of emulsifying agent and time for stirring were varied in preliminary trial batches from 1-3 % v/v and 1-3 h, respectively. In factorial design batches J1- J9, 2.0 % v/v Span 80 was used as an emulsifying agent and time for stirring was kept to 3 h. The drug-to-polymer-to-polymer ratio and stirring speed were varied in batches J_1_- J_9_, as shown in Table 1. All other variables were similar to the preliminary trial batches. Microspheres thus obtained were filtered and washed several times with petroleum ether (80:20) to remove traces of oil. The microspheres were then dried at room temperature (25 ^o^C and 60 % RH) for 24 h. The effect of formulation variables on characteristics of the microspheres of factorial design batches has been summarized in Table 1.

**Table 1:** Various batches of propranolol hydrochloride mucoadhesive microspheres, prepared using the 32 full factorial design layout

Batch code	Variable levels		*In-vitro *wash- off test (% mucoadhesion after 1 h)		Drug entrapment efficiency (%)		Particle size (μm)		t_80_ (min)
	X_1_	X_2_							
J_1_ J_2_ J_3_ J_4_ J_5_ J_6_ J_7_ J_8_ J_9_	-1 -1 -1 0 0 0 1 1 1	-1 0 1 -1 0 1 -1 0 1	58 56 42 82 78 70 92 80 74		25 20 29 54 51 40 45 40 33		100 95 88 110 103 96 115 111 102		589 640 720 496 537 579 294 306 333
Translation of coded levels in actual units									
Variable levels				Low (-1)		Medium (0)		High (+1)	
Drug-to-polymer-to polymer ratio (X_1_) (Propranolol hydrochloride-ethyl cellulose-Carbopol-934P) Stirring speed (X_2_) rpm				1:3:0.5 800		1:3:1 1000		1:3:1.5 1200	
All the batches were prepared using 2 % v/v Span 80 and a stirring time of 3 h.									

*Optimization of microspheres formulation using 32 full factorial design*

A statistical model incorporating interactive and polynomial terms was utilized to evaluate the responses. 

Y = b_0_ + b_1_X_1_ + b_2_X_2_ + b_12_X_1_X_2_ + b_11_X_1_^2^ + b_22_ X_2_^2^                     (1)

Where *Y *is the dependent variable, b_0_ is the arithmetic mean response of the nine runs, and b_i _is the estimated coefficient for the factor *X*_i_. The main effects (*X*_1_ and *X*_2_) represent the average result of changing one factor at a time from its low to high value. The interaction terms (*X*_1_*X*_2_) show how the response changes when two factors are simultaneously changed. The polynomial terms (*X*_1_^2^ and *X*_2_^2^) are included to investigate non-linearity. On the basis of the preliminary trials, a 32 full factorial design was employed to study the effect of independent variables, i.e. drug-to-polymer-to-polymer (*X*_1_) and the stirring speed (*X*_2_) on dependent variables which were the percentage of mucoadhesion, drug entrapment efficiency, particle size and the time required for 80 % drug dissolution (t_80_). 

*Determination of propranolol hydrochloride*

The amount of propranolol hydrochloride was estimated, using a UV/Vis spectrophotometric method (Shimadzu UV-1700 UV/Vis double beam spectrophotometer, Kyoto, Japan). Aqueous solutions of propranolol hydrochloride were prepared in 0.1 N hydrochloride acid (pH 1.2) and absorbance was measured on a Shimadzu UV/Vis spectrophotometer at 289 nm. The method was validated for linearity, accuracy and precision. The method obeyed from the Beer’s Law in the concentration range of 10 to 50 μg/mL. When a standard drug solution was analyzed repeatedly (n = 5), the mean error (accuracy) and relative standard deviation (precision) were found to be 0.84% and 1.2%, respectively.

*Drug entrapment efficiency*

Two hundred milligrams of accurately weighed microspheres were crushed in a glass mortar and the powdered microspheres were suspended in 10 mL of 0.1 N hydrochloric acid (pH=1.2). After 24 h, the solution was filtered and the filtrate was analysed for the drug content. The drug entrapment efficiency was calculated using the following formula: Practical drug content/Theoretical drug content × 100. The drug entrapment efficiency for batches J_1_-J_9_ has been reported in Table 1.

*Particle size of microspheres*

The particle size of the microspheres was determined, using an optical microscopy method (22). Approximately 300 microspheres were counted for particle size, using a calibrated optical microscope (Labomed CX RIII, Ambala, India). The particle size of microspheres of batches J_1_-J_9_ has been reported in Table 1. 

*In-vitro wash-off test for microspheres*

The mucoadhesive properties of the microspheres were evaluated, using an *in-vitro *wash-off test, as reported by Lehr et al (23). A 1x1 cm piece of rat stomach mucosa was tied onto a glass slide (3 inch-by-1 inch), using thread. Microspheres were spread (approximately 50) onto the wet rinsed tissue specimen and the prepared slide was hung onto one of the groves of a USP tablet disintegration test apparatus, with continuous oxygen supply. The disintegration test apparatus was operated, giving the tissue specimen was given regular up and down movements within the beaker of the disintegration apparatus, which contained the simulated gastric fluid (pH=1.2). At the end of 30 min, 1 h and at hourly intervals up to 12 h, the number of microspheres still adhering onto the tissue was counted. The results of *in-vitro *wash-off test of batches J_1_-J_9_ have been shown in Table 1.

*Scanning electron microscopy*

**Figure 1:**
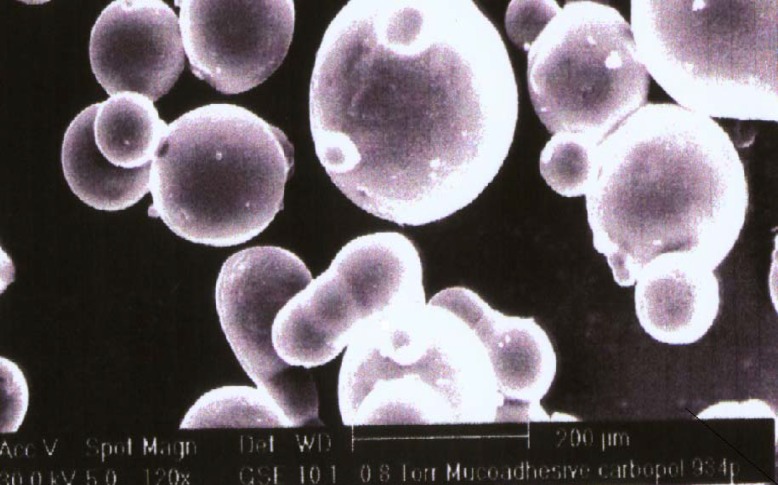
Scanning electron photomicrograph of propranolol hydrochloride loaded Carbopol-934P mucoadhesive microspheres (batch J_4_).

Scanning electron photomicrograph of drug-loaded Carbopol-934P mucoadhesive microspheres were taken. A small amount of microspheres was spread on a glass stub. Afterwards, the stub containing the sample was placed in the scanning electron microscope (JSM 5610 LV SEM, JEOL, Datum Ltd, Tokyo, Japan) chamber. A scanning electron photomicrograph was then taken at an acceleration voltage of 20 KV, and a chamber pressure of 0.6 mm Hg, at different magnifications. The photomicrograph of batch J_4 _has been depicted in Figure 1. 

**Figure 2:**
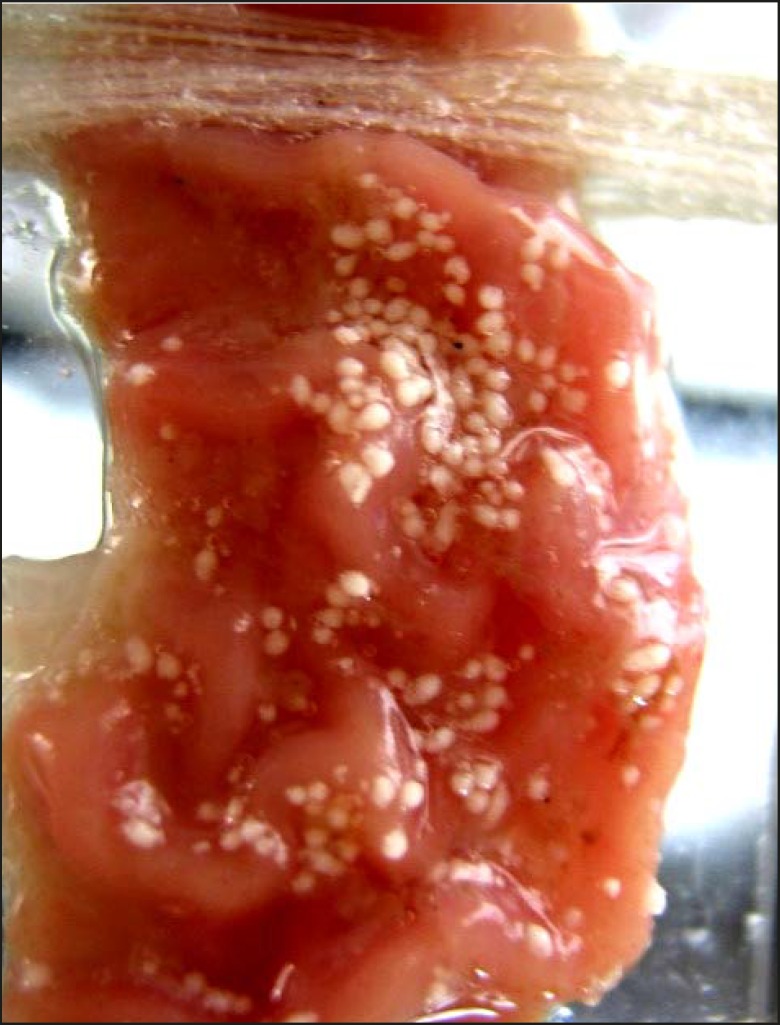
*In-vitro *wash-off test carried out on propranolol hydrochloride loaded Carbopol-934P mucoadhesive microspheres (batch J_4_), using rat stomach, after 1 h

**Figure 3:**
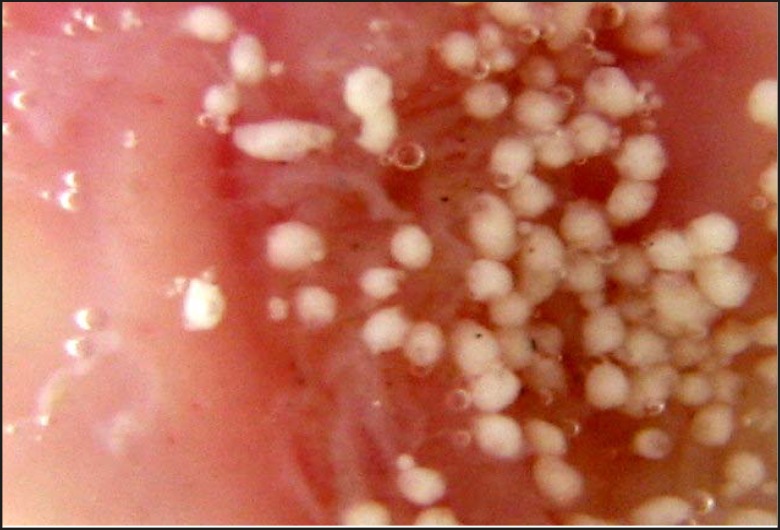
*In-vitro *wash-off test carried out on propranolol hydrochloride loaded Carbopol-934P mucoadhesive microspheres (batch J_4_ ), using rat stomach, after 8h

The photomicrograph of the *in-vitro *wash-off test after 1 h and 8 h have been depicted in Figures 2 and 3, respectively.

*Drug release study*

**Figure 4:**
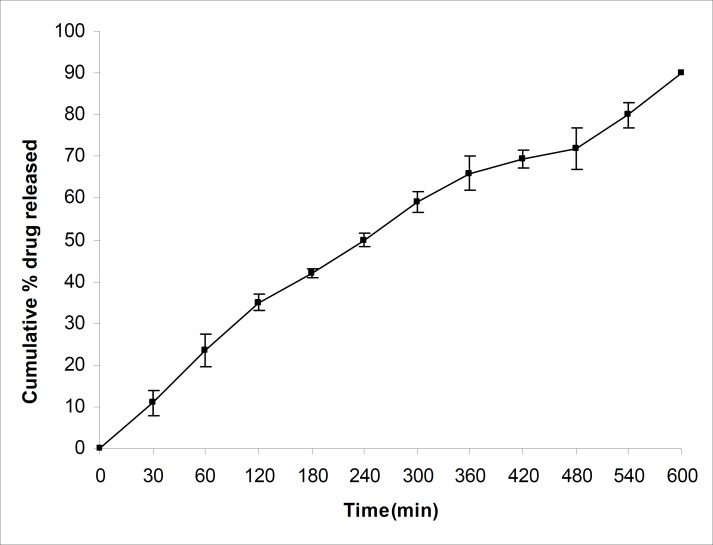
*In-vitro *dissolution of propranolol hydrochloride from batch J_4_ mucoadhesive microspheres in 0.1 N hydrochloric acid (pH= 1.2) (n=3, mean **±**SD).

The drug release studies were carried out using a USP XXIV basket apparatus (Electrolab, TDT-06T, India) at 37°C ± 0.5°C and at 100 rpm, within 900 mL of 0.1 N hydrochloric acid (pH=1.2) as the dissolution medium, as per USP XXVI dissolution test described for propranolol hydrochloride tablets. Microspheres equivalent to 40 mg of propranolol hydrochloride were used for this purpose. Five milliliters of the sample solution was withdrawn at predetermined time intervals, filtered through a 0.45 μm membrane filter, diluted suitably and analysed spectrophotometrically at 289nm. An equal amount of fresh dissolution medium was replaced immediately after withdrawal of the test sample. Percentage of drug dissolved at different time intervals was calculated, using the Beer-Lambert equation. The t_80_ was calculated, using the Weibull equation (24). The average values of t80 for batches J_1_-J_9_ have been shown in Table 1. The percentage of drug released from batch J_4 _in pH 1.2 has been shown in Figure 4. 

*Data fitting*

An attempt was made to fit the dissolution data into the Hixon-Crowell (24) model, represented as: 

m = [(100) * (1/3) – k * t] ^3                    ^(2)

Where k is the Hixon-Crowell constant [mass/ time]^1/3^. In this model the percentage of unreleased drug versus cube root of time is linear.

The dissolution data were also treated with the Korsmeyer-Peppas model (25), in order to characterize the mechanism of drug release:

M_t_ / M_∞_ = K_p_ n                     (3) 

M_t_/M_∞_ represents the fraction of drug released at time t and Kp is the kinetic constant characterizing the polymeric system and “n” stands for the diffusion exponent.

The dissolution data were also analyzed, using the Weibull equation (24), in an attempt to determine the kinetics of drug release from different batches of mucoadhesive microspheres:

m = 1- exp [-(t-t_i_ ) b/a]                     (4)

Where “a” is the scale parameter which defines the time scale of the process, t_i_ is the location parameter which represents the lag period before the actual onset of dissolution process (in most cases t_i_ = 0) and “b” is the shape parameter. In this model, the plot of log of time versus In (1-m) is linear.

The results of *F*-statistics were used for the selection of the most appropriate model. Results of *F*-statistics and the summary of results of regression analysis are shown in Tables 2 and 3, respectively.

**Figure 5:**
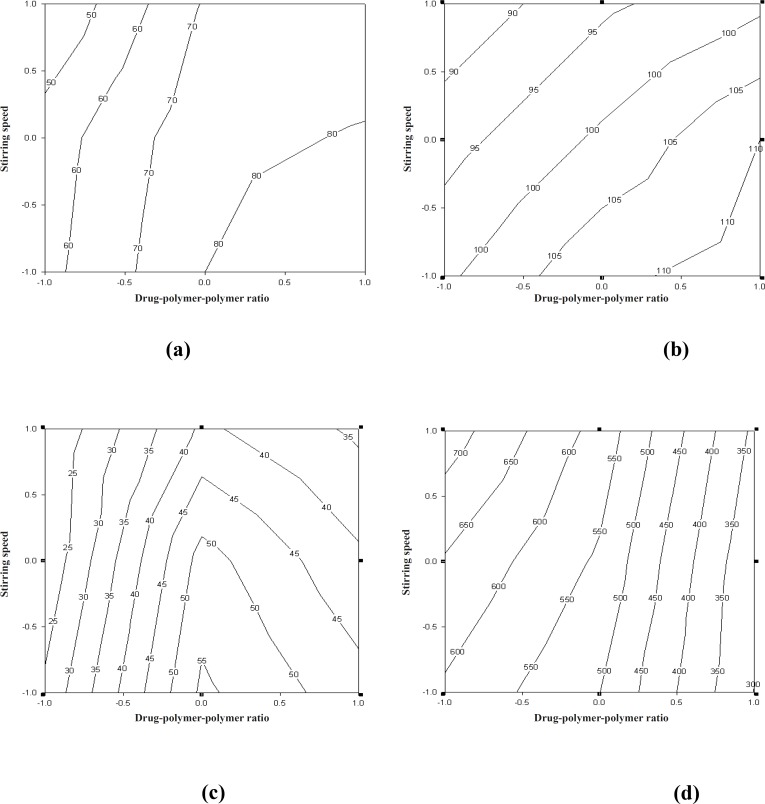
Counter plot showing the effect of drug-polymer-polymer ratio (X_1_) and stirring speed (X_2_) on: % mucoadhesion (a), particle size (b), drug entrapment efficiency (c) and t_80_ (d).

The curve fitting, simulation and plotting were performed, using the Excel software (Microsoft Software Inc., USA) and Sigma plot^®^ version 10.0 (Sigma plot software, Jangel Scientific Software, San Rafael, CA). The effects of independent variables on the response parameters were visualized from the contour plots. Numerical optimization, using the desirability approach, was employed to locate the optimal settings of the formulation variables so as to obtain the desired response (26). An optimized formulation was developed by setting constraints on the dependent and independent variables. The formulation developed was evaluated for the responses, and the experimental values obtained were compared with those predicted by the mathematical models generated. Counter plots showing the effect of drug-to-polymer-to-polymer (*X*_1_) and stirring speed (*X*_2_) on the percentage of mucoadhesion, drug entrapment efficiency, particle size and t_80_ have been shown in Figure 5. 

*In-vivo study*

**Figure 6:**
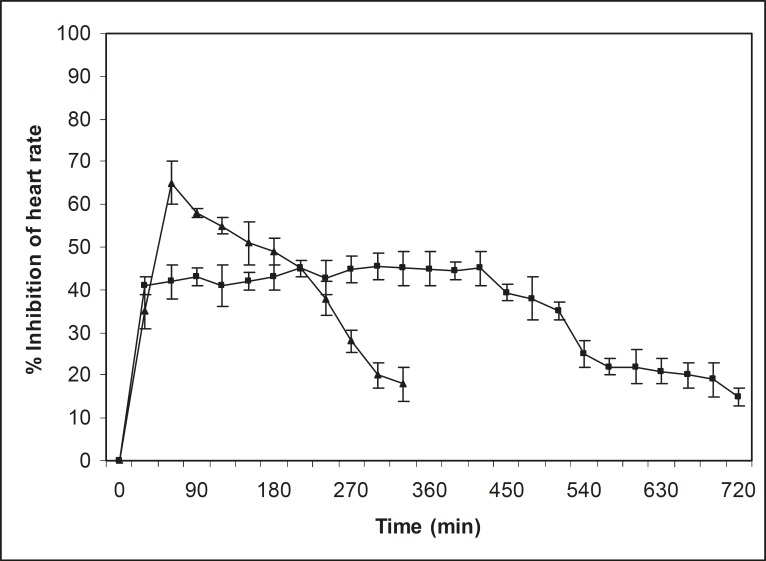
Percentage of inhibition of heart rate, after oral administration of propranolol hydrochloride (-▲-) and its batch J_4_ mucoadhesive microspheres (-■-) in rabbits. (n= 3, mean ± SD)

*In-vivo *studies on propranolol hydrochloride mucoadhesive microspheres were performed on normal healthy Indian white rabbits (lupas) of mixed sex, weighing 2.3 to 2.65 kg each. The approval of the Institutional Animal Ethics Committee was obtained, before starting the study. The study was conducted in accordance with the standard intuitional guidelines. Two groups of normal healthy rabbits (three in each group) that were fasted (with water) at least 12 h before studies, were used for this investigation. Before oral drug administration of respective dosage forms, normal heart rate was recorded for 15 min. After recording the normal heart rate, i.v. isoprenaline (0.25 μg/kg) was administrated for induction of heart rate at a fixed interval. A dose of 2.5 mg/kg of propranolol hydrochloride-containing mucoadhesive microspheres and propranolol hydrochloride powder were administrated orally using long food needles. After oral administration of dosage forms, heart rate was continuously recorded for 12 h, using pulse transducer (MI) through a Powerlab-multichannel computerized data acquisition system (AD Instruments, Australia) from each rabbit. The percentage of inhibition of heart rate was measured and results have been shown in Figure 6. 

## Results and Discussion

*Preliminary trials*

The mucoadhesive propranolol hydrochloride microspheres prepared from Carbopol-934P and ethyl cellulose were made using the emulsion-solvent evaporation technique. Carbopol-934P chosen for the preparation of mucoadhesive microspheres, owing to its good mucoadhesive properties. Ethyl cellulose was used as a carrier polymer. Different concentrations of span 80, from 1-3% v/v, were used as the emulsifying agent. Span 80 was found to have a significant influence on the percentage of mucoadhesion observed (i.e. percentage of microspheres adhered and remained on the gastric mucous layer), particles size and drug entrapment efficiency. Results showed that increase in the concentration of span 80, increased the particle size of microspheres, as well as the percentage of mucoadhesion. However, the drug entrapment efficiency was decreased. At a 1% v/v span80 concentration, percentage of mucoadhesion, particle size and drug entrapment efficiency of microspheres were 65%, 82 μm and 64 % respectively. However, formation of irregularly shaped microspheres was observed. On the other hand, at 3% v/v span 80 concentration, the percentage of mucoadhesion, particles size and drug entrapment efficiency of microspheres were 80%, 208 μm and 39 % respectively. The shapes of microspheres were found to be spherical, particles were coalesced. However, a 2 % v/v of concentration of span 80 was used for further studies. 

One of the important factors related to microspheres, as reported by Lee *et al *(27), is the viscosity of the polymer solution. Polymer concentrations of 0.5%, 1%, and 2% w/v were selected for preliminary trials. Flake formation was observed when ethyl cellulose and Carbopol-934P concentration was used at a level of 0.5% w/v, whereas maximum sphericity was observed at the 1% w/v level. Non-spherical microspheres were formed, when 2% w/v using polymer concentrations. Therefore, a 1% w/v concentration of ethyl cellulose and Carbopol-934P, each in ethanol, was found to be the optimum concentration for the polymer solution. A 1:1 mixture of heavy and light liquid paraffin was found to be suitable as the dispersion medium. 

Preliminary trial batches were prepared, in order to investigate the effect of stirring time and speed on the percentage of mucoadhesion, drug entrapment efficiency, and characteristics of the resulting microspheres. An increase in the stirring time 1 h to 3 h, showed an increase in the percentage of mucoadhesion, but a decrease in drug entrapment efficiency and particles size of microspheres. Thus, a stirring time 3h was selected for further studies. Since, the stirring speed had a significant effect on the percentage of mucoadhesion, drug entrapment efficiency and particles size of microspheres, it was selected as an important factor for further studies. 

On the basis of the preliminary trials, a 3^2^ full factorial design was employed to study the effect of independent variables (i.e. drug-to-polymer-to-polymer ratio [*X*_1_] and the stirring speed [*X*_2_]) on dependent variables, which were the percentage of mucoadhesion, drug entrapment efficiency, particle size and t_80_. The results depicted in Table 1 clearly indicate that all the dependent variables are strongly dependent on the selected independent variables, as they show a wide variation among the nine batches (J_1_-J_9_). The fitted equations (full models), relating the responses (i.e. percentage of mucoadhesion, drug entrapment efficiency, particle size and t_80_) to the transformed factor are shown in Table 3. The polynomial equations can be used to draw conclusions after considering the magnitude of coefficient and the mathematical sign it carries (i.e. positive or negative). The high values of correlation coefficient (Table 3) for the dependent variables indicate a good fit. The equations may be used to obtain estimates of the response, since a small error of variance was noticed in the replicates.

**Table 3:** Summary of the regression analysis results

**Coefficient**	**b**_0_	**b**_1_	**b**_2_	**b**_11_	**b**_22_	**b**_12_	**R** ^2^
% Mucoadhesion	77.42	14.47	-8.19	-2.81	-9.60	-0.50	0.9809
Drug entrapment efficiency	48.14	7.71	-3.28	-4.57	-16.71	0.28	0.9165
Particle size	103.77	7.30	-6.60	-1.10	-1.01	-1.25	0.9955
t_80_	533.41	-167.50	43.88	-46.19	3.18	-23.49	0.9997

*Factorial equation for the percentage of mucoadhesion*

The *in-vitro *mucoadhesiveness test represented the percentage of mucoadhesive microspheres remaining on the stomach mucosa (Table 1). The mucoadhesive microspheres of all the of the factorial design batches were spherical (Figure 1, batch J_4_) and free-flowing. 

The linear model generated for the percentage of mucoadhesion was found to be significant, with an *F*-value of 20.64 (p < 0.0001) and R^2^ value of 0.9809:

% mucoadhesion = 77.42+ 14.47*X*_1_ – 8.19*X*_2_ -2.81*X*_1_*X*_2 _-9.6 *X*_2_^2                    ^ (5)

The counter plot (Figure 5a) shows that the in-vitro wash-off test carried out for determining the percentage of mucoadhesion of microspheres, increased from 42 to 58 and 74 to 92, at lower and higher levels of drug-to-polymer-to-polymer ratio, respectively, as the stirring speed decreased. Results obtained, indicated that the effect of *X*_1_ (drug-to-polymer-to-polymer) is more significant than *X*_2_ (stirring speed). Moreover, stirring speed had a negative effect on the percentage of mucoadhesion (i.e. as the stirring speed increased, the percentage of mucoadhesion decreased). This finding may be attributed to a change in particle size, which can consequently affect mucoadhesion. As the drug-to-polymer-to-polymer ratio increases; the percentage of mucoadhesion also increases; since the greater amount of polymer results in a higher amount of free –COOH (carboxyl) groups (17), which are responsible for binding to the sialic acid groups within the mucus network. Hence, it results in an increase in the mucoadhesive properties of microspheres. *In-vitro *mucoadhesive tests showed that propranolol hydrochloride mucoadhesive microspheres adhered more strongly to the gastric mucosa and could be retained in the gastrointestinal tract for an extended period of time (Figures 2 and 3). Figure 3 also showed that even after 8 h some of the microspheres were remained on the gastric mucous layer. 

*Factorial equation for particle size*

The linear model generated for particle size of microspheres was found to be significant, with an *F*-value of 88.76 (p < 0.0001) and R^2^ value of 0. 9955: 

Particle size = 103.77 + 7.3*X*_1_ – 6.6*X*_2_ -1.0*X*_1_*X*_2_ -1.0 *X*_2_^2                    ^(6)

The counter plot (Figure 5b) showed that the particle size of microspheres increased from 88 to 100 μm and 102 to 115 μm, at lower and higher levels of drug-to-polymer-to-polymer ratio, respectively, as the stirring speed decreased. The results obtained indicate that the effect of *X*_1 _(drug -to-polymer-to-polymer) is more significant than *X*_2_ (stirring speed). This means that, as the stirring speed increases, the particle size decreases, and as a result the percentage of mucoadhesion could be directly affected. 

Thus, one can conclude that the amount of polymer (Carbopol-934P) and the stirring speed directly affect the percentage of mucoadhesion, as well as the particles size of microspheres.

*Factorial equation for drug entrapment efficiency*

The drug entrapment efficiency and t_80 _are important variables for assessing the drug loading capacity of microspheres and their drug release profile. These parameters are dependent on the process of preparation, physicochemical properties of drug, and formulation variables. 

The linear model generated for drug entrapment efficiency was found to be significant, with an *F*-value of 4.39 (p < 0.0001) and R^2^ value of 0.9165:

Drug entrapment efficiency = 48.14 + 7.71*X*_1_ -3.28*X*_2_ -4.57*X*_1_*X*_2 _-16.71*X*_1_^2^ +0.28 *X*_2_^2^                     (7)

The counter plot (Figure 5c) shows that the percentage of drug entrapment efficiency of microspheres increased from 25.0 to 29.0 and 33.0 to 45.0, at lower and higher levels of drug-to-polymer-to-polymer ratio, respectively, as the stirring speed decreased. However, at a medium level of drug-to-polymer-to-polymer ratio, as the stirring speed decreased, the percentage of drug entrapment efficiency of microspheres showed an increase from 40.0 to 54.0. The results obtained, indicate that the effect of *X*_1_ (drug-to-polymer-to-polymer) is more significant than *X*_2_ (stirring speed). Moreover, the stirring speed had a negative effect on the percentage of drug entrapment efficiency (i.e. as the stirring speed increased, the particle size decreased and consequently the drug entrapment efficiency also decreased).

*Factorial equation for t*_80_

The linear model generated for t_80_ was found to be significant, with an *F*-value of 115.91 (p < 0.0001) and R^2^ value of 0.9997:

t_80_ = 533.47 – 167.16*X*_1_+ 43.88*X*_2_ -25.57*X*_1_*X*_2_ -58.71*X*_1_^2^ +5.57 *X*_2_^2^                     (8)

The counter plot (Figure 5d) shows that the percentage of drug released *in-vitro *from microspheres decreased at the lower and higher levels of drug-to-polymer-to-polymer ratio, respectively, as the stirring speed decreased. The results depicted in Table 3 indicate that the percentage of drug released *in-vitro *is highly dependent on the drug-to-polymer-to-polymer ratio and the stirring speed. The drug-to-polymer-to-polymer ratio has a negative effect on t_80_, while stirring speed has a positive effect on t_80_. Consequently, as the particle size decreases, the drug release also decreases. 

A numerical optimization technique, using the desirability approach, was employed to develop a new formulation with the desired responses. Constraints like maximizing the percentage of mucoadhesion, drug entrapment efficiency, particle size and the amount of drug released after 12 h, in addition to minimizing the t_80_, were set as goals to locate the optimum settings of independent variables in the new formulation. The optimized microsphere formulation (J_10_) was developed, using a 1:3:1.25 drug-to-polymer-to-polymer ratio and a stirring speed of 950 rpm. The optimized formulation was evaluated for the percentage of mucoadhesion, drug entrapment efficiency, particle size and the amount of drug released. The results of experimentally observed responses and those predicted by mathematical models, along with the percentage prediction errors were compared. The prediction error, for the response parameters ranged between 0.52 and 2.18%, with an absolute error value of 1.26 ± 0.72%. The low values of error indicate the high prognostic ability of factorial equation methodology. The amount of drug released from the optimized formulation was found to be low and it had a t_80_ value of 405 min. Thus, batch J_4_ was selected for further studies, since it exhibited a high t_80_ value of 496 min and seems to be a promising candidate for achieving drug release up to 12 h. The drug release profile of batch J_4_ is shown in Figure 4. This graph revealed that drug release rate slowed down after 2 h. 

The results of curve fitting of the best batch into different mathematical models are given in Table 2. 

**Table 2:** Results of the mathematical models fitted on batch J_4_

	**Hixon-Crowell**	**Korsemeyer and Peppas**	**Weibull**
SSR	95.11	158.47	57.24
F-value	11.62	22.39	8.05
Correlation Coefficient	0.9871	0.9825	0.9931
Slope	0.005	0.947	1.210
Intercept	–0.99	–2.36	–2.12

The mechanism of drug release from the microspheres was found to be diffusion controlled, since the plots of the cumulative percentage of drug release versus the square root of time were found to be linear with the regression coefficient (R^2^) values, ranging from 0.9784 to 0.9879 for the best batch. The release profile fitted the Weibull equation, and an *F*-value of 8.05 was obtained. The value obtained for the correlation coefficient was found to be 0.9931. The values of slope and intercept were found to be 1.21 and –2.12, respectively. The release profile fitted to the Korsmeyer-Peppas equation, produced an *F*-value of 22.39. The value of correlation coefficient was found to be 0.9825. The values of slope and intercept were found to be 0.947 and –2.36, respectively. Finally, the release profile fitted to the Hixon-Crowell equation, gave an *F*-value of 11.62. The value of correlation coefficient was found to be 0.9871. The values of slope and intercept were found to be 0.005 and –0.99, respectively. The results of *F*-statistics were used for the selection of the most appropriate model. As a result, it was concluded that the release profile fitted best to the Weibull equation (*F*-value =8.05).

*In-vivo studies*

A rapid reduction in heart rate was observed with pure propranolol hydrochloride and the heart rate also recovered rapidly to the normal level within 5 h (Figure 6). In the case of propranolol hydrochloride mucoadhesive microspheres, the reduction in heart rate was slow and reached a maximum reduction of 47 percent within 5 h after oral administration. This reduction in heart rate was sustained over a longer period of time (10 h). The 40 percent reduction in heart rate could be considered as a significant anti-hypertensive effect (28). In pure drug, the significant anti-hypertensive effect (40 percent) was maintained during the periods from 0.5 to 5 h following oral administration of propranolol hydrochloride. Whereas, in case of mucoadhesive microspheres, the effect was maintained for a period of 0.5 to 10 h. The sustained anti-hypertensive effect observed over a longer period of time in case of mucoadhesive microspheres was due to a slow release rate of drug, as well as the mucoadhesive properties of microspheres. 

In conclusion, it could be said that the propranolol hydrochloride mucoadhesive microspheres developed, using a 3^2^ full factorial design, showed a high percentage of mucoadhesion and drug entrapment efficiency. They also exhibited a sustained drug release property for peroral use in the form of capsule. Drug-to-polymer-to-polymer (propranolol hydrochloride-ethyl cellulose-Carbopol-934P) ratio, as well as the stirring speed had a significant influence on the percentage of mucoadhesion, drug entrapment efficiency, particle size and t_80_. The optimized propranolol hydrochloride mucoadhesive microsphere formulation, developed using the desirability approach, showed a greater anti-hypertensive effect over a period of 10 h, compared to the propranolol hydrochloride powder. This would indicate the potential of mucoadhesive propranolol hydrochloride microspheres for use in the provision of a sustained anti-hypertensive effect. 
